# Supplementary material for: Convenience Food Options and Adequacy of Nutrient Intake among School Children during the COVID-19 Pandemic
Source: Nutrients. 2022 Jan 31;14(3):630. doi: 10.3390/nu14030630 (PMC8838923; doi:10.3390/nu14030630)
Supplement: Supplementary file 1 [file nutrients-14-00630-s001.zip › nutrients-1529421-supplementary.pdf]

Table S1. Crude and multinomial regressions for significant SES determinants and literacy scores.

		Relative Risk (CI 95%)			
		**Adequate**	**Excess**	**Deficient**	**Inadequate**
Instant Food (*n* = 666)	Crude (excluding missing income)	1.00	2.3 (1.3, 4.2) **	2.7 (0.7, 11.1)	3.0 (1.6, 5.6) **
	Crude (all sample)	1.00	2.5 (1.4, 4.4) **	2.7 (0.7, 10.7)	3.4 (1.9, 6.2) ***
	Model 2 (gender + grade + family size)	1.00	2.6 (1.5, 4.6) **	3.2 (0.8, 13.0)	3.3 (1.8, 6.1) ***
	Model 3 (gender + grade + income)	1.00	2.2 (1.2, 4.0) **	3.0 (0.7, 12.5)	2.4 (1.2, 4.5) **
	Model 4 (gender + grade + father education)	1.00	2.6 (1.5, 4.6) **	3.4 (0.8, 14.4)	3.0 (1.6, 5.6) **
	Model 5 (gender + grade + mother education)	1.00	2.5 (1.4, 4.3) **	2.8 (0.7, 11.2)	3.1 (1.7, 5.7) ***
	Model 6 (gender + grade + skill)	1.00	2.2 (1.3, 3.9) **	2.7 (0.7, 11.1)	2.4 (1.3, 4.5) **
	Model 7 (gender + grade + attitude)	1.00	2.4 (1.4, 4.2) **	2.8 (0.7, 11.4)	2.6 (1.4, 4.9) **
Frozen Food (*n* = 660)	Crude (excluding missing income)	1.00	1.3 (0.9, 2.0)	1.6 (0.5, 5.4)	1.4 (0.8, 2.2)
	Crude (all sample)	1.00	1.3 (0.9, 1.9)	1.4 (0.5, 4.6)	1.4 (0.9, 2.2)
	Model 2 (gender + grade + family size)	1.00	1.3 (0.9, 2.0)	1.4 (0.4, 4.5)	1.3 (0.9, 2.1)
	Model 3 (gender + grade + income)	1.00	1.3 (0.9, 2.0)	1.6 (0.5, 5.5)	1.4 (0.8, 2.2)
	Model 4 (gender + grade + father education)	1.00	1.3 (0.9, 2.0)	1.6 (0.5, 5.4)	1.5 (0.9, 2.4)
	Model 5 (gender + grade + mother education)	1.00	1.3 (0.9, 1.9)	1.4 (0.4, 4.6)	1.4 (0.9, 2.2)
	Model 6 (gender + grade + skill)	1.00	1.2 (0.8, 1.8)	1.2 (0.4, 4.1)	1.1 (0.7, 1.8)
	Model 7 (gender + grade + attitude)	1.00	1.2 (0.8, 1.8)	1.3 (0.4, 4.2)	1.3 (0.8, 2.1)
Take-Out Food (*n* = 663)	Crude (excluding missing income)	1.00	1.2 (0.8, 1.8)	0.7 (0.2, 2.7)	2.1 (1.3, 3.3) **
	Crude (all sample)	1.00	1.2 (0.8, 1.7)	0.6 (0.2, 2.4)	2.0 (1.3, 3.1) **
	Model 2 (gender + grade + family size)	1.00	1.2 (0.8, 1.7)	0.6 (0.2, 2.4)	2.0 (1.3, 3.1) **
	Model 3 (gender + grade + income)	1.00	1.2 (0.8, 1.8)	0.7 (0.2, 2.7)	1.8 (1.1, 2.9) *
	Model 4 (gender + grade + father education)	1.00	1.1 (0.8, 1.7)	0.7 (0.2, 2.6)	1.9 (1.2, 2.9) **
	Model 5 (gender + grade + mother education)	1.00	1.2 (0.8, 1.7)	0.5 (0.1, 2.1)	1.8 (1.2, 2.8) **
	Model 6 (gender + grade + skill)	1.00	1.1 (0.7, 1.6)	0.6 (0.2, 2.2)	1.6 (1.0, 2.6) *
	Model 7 (gender + grade + attitude)	1.00	1.1 (0.8, 1.6)	0.6 (0.1, 2.2)	1.6 (1.0, 2.6) *

[1] * $p < 0.05$, ** $p < 0.01$, *** $p < 0.001$.

Table S2. Associations between convenience food intake and missing income data.

		Relative Risk (CI 95%)				
		**Adequate**	**Excess**	**Deficient**	**Inadequate**	***p*-Value**
Among those with data for instant foods	With income data	157 (27.4%)	12 (2.1%)	266 (46.3%)	139 (24.2%)	0.8492
	Without income data	23 (25.0%)	1 (1.1%)	46 (50.0%)	22 (23.9%)	
Among those with data for frozen foods	With income data	154 (27.1%)	12 (2.1%)	264 (46.4%)	139 (24.4%)	0.8829
	Without income data	23 (25.3%)	1 (1.1%)	45 (49.5%)	22 (24.4%)	
Among those with data for take-out foods	With income data	157 (27.5%)	11 (1.9%)	264 (46.2%)	139 (24.3%)	0.8646
	Without income data	23 (25.0%)	1 (1.1%)	46 (50.0%)	22 (23.9%)	
Among all people with nutrient data	With income data	158 (27.4%)	12 (2.1%)	268 (46.4%)	139 (24.1%)	0.8237
	Without income data	23 (24.5%)	1 (1.1%)	47 (50.0%)	23 (24.5%)	
